# Physical activity and sedentary behaviour of adolescents and their parents: a specific analysis by sex and socioeconomic status

**DOI:** 10.1186/s13690-023-01185-1

**Published:** 2023-10-24

**Authors:** Eduardo Duarte de Lima Mesquita, William Rodrigues Tebar, Dayane Cristina Queiroz Correia, Juziane Teixeira Guica, Wésley Torres, Rômulo Araújo Fernandes, Ricardo Ribeiro Agostinete, Diego Giulliano Destro Christofaro

**Affiliations:** 1 Laboratory of InVestigation in Exercise – LIVE, Department of Physical Education, São Paulo State University (UNESP), Presidente Prudente, São Paulo, Brazil; 2 Post-Graduation Program in Movement Sciences, Institute of Biosciences, São Paulo State University (UNESP), São Paulo, Brazil; 3 Center of Clinical and Epidemiological Research, University Hospital, University of Sao Paulo (USP), São Paulo, Brazil

**Keywords:** Sedentary Behaviour, Physical activity, Adolescence, Childhood, Family

## Abstract

**Background:**

The association of lifestyle habits of parents and of their children has been widely investigated as an important determinant for healthy habits in youth. Although parental sociodemographic characteristics are potential confounding factors in parent-child physical activity (PA) and sedentary behaviour (SB), it is still unclear whether these factors have a moderating role in this association. This study aimed to analyze the association of parent-child PA and SB according to parental sex and economic level in adolescents.

**Methods:**

The study sample was made up of 1231 adolescents (15.6 ± 1.1 years, 58.2% of girls), 1202 mothers and 871 fathers. The leisure-time and commuting PA was assessed by the Baecke questionnaire, while sedentary behaviour (SB) was assessed according to hours per week of television viewing and computer use, by both adolescents and their parents. Economic status was analyzed using a questionnaire and classified as low, medium, and high. Linear models were used to assess the association of parent-child PA and SB in the different domains according to parental sex and economic level.

**Results:**

Leisure time was associated between boys and their fathers in high (β = 0.23, p = 0.044) and low economic classes (β = 0.31, p < 0.001), and girls and their mothers in low economic class (β = 0.38, p < 0.001). Commuting PA was associated between adolescents and both parents in low economic class (fathers β = 0.21, p = 0.005; mothers (β = 0.15, p = 0.020). TV time of boys was associated with TV time of fathers in low economic class (β = 0.13, p = 0.022) and with TV time of mothers in medium economic class (β = 0.13, p = 0.046). Among girls, TV time was associated with TV time of both parents only in low economic class (fathers β = 0.28, p < 0.001; mothers β = 0.25, p < 0.001). Computer use of girls was associated with computer use of fathers in high economic class (β = 1.72, p = 0.043) and mothers in low economic class (β = 0.57, p = 0.014), while no association was observed among boys.

**Conclusion:**

Economic status was shown to be an important moderator of the association between parent-child PA and SB in adolescents.

**Table:** 

**Text box 1. Contributions to the literature**
• Most studies analyzing the relationship between lifestyle habits between parents and children have been carried out in ‘developing’ countries, this study will contribute with data from Brazil;
• This study explored in a stratified way the relationship between parents and their children, considering the different socioeconomic conditions and the sex, showing which segments should have priorities for increasing physical activity and decreasing sedentary behaviour in the future;
• Low economic parents, apparently, influence more on time spent at physical activity and/or sedentary behaviour in their children, showing that actions to promote physical activity and reduce sedentary behaviour should focus on low-income populations.

## Background

Sedentary behaviour (SB) is defined as activities of energy expenditure ≤ 1.5 metabolic equivalent units (METs), spent in a seated, reclined, or lying posture during awake time [1]. A worldwide increase in the prevalence of SB has been observed, mainly due to behavioural changes and technological advances in modern society, both at work (e.g., using computer) and in leisure time (e.g., watching TV) [2]. High amounts of SB are associated with several health problems in different age groups, including children and adolescents [3, 4]. In addition, SB values during childhood and adolescence are important determinants of paediatric obesity, [5] which increases the risk of obesity [6] and morbidities [7] in adulthood.

Alternatively, regular practice of physical activity (PA) during childhood and adolescence has been shown to be a protective factor for obesity and disease occurrence throughout life, mainly regarding leisure-time PA, such as exercise/sport participation [8, 9]. This protection seems to occur through the control of body fat, mainly due to maintenance of the practice of leisure-PA throughout life (tracking of physical activity) [10], in which youth practice of PA predicts PA in adulthood. This can be justified by some hypotheses, such as “carry-over value”, “ability and readiness”, and “habit formation hypothesis” [11]. Furthermore, besides leisure-time PA, commuting PA has been associated with improved cardiovascular health, body composition, and physical fitness [12, 13].

In this sense, considering the importance of PA and SB for paediatric health, the literature has made efforts to understand their determinants and associated factors in adolescents. These determinants include biological, cognitive, and behavioural variables, as well as which, findings in the literature show that social aspects may represent an important role for children’s PA, such as the influence of parents [14]. The theory proposed by Albert Bandura [15] explains the parent-child interaction on human motivation and action in a reciprocal causation integrating behavioural, environmental and cognitive factors for personal and social change, while Welk [16] specifically presents parental aspects as determinants of children’s physical activity through a social-ecological framework with input of personal, social and environmental influences [14]. Among these parental aspects, parents’ habits and lifestyle and the influence of these aspects on sedentary behaviour and levels of PA of children and adolescents have been widely studied.

The literature shows a significant association between parents’ PA/SB and children’s PA/SB, however, most of the evidence on the subject is concentrated in developed countries [14, 17]. Furthermore, some factors can affect this association, such as the sex of the parents and children [18] and parental socioeconomic status [19]. Of note, Brazil is a developing country of continental dimension with high income inequality, generating specific and distinct socio-demographic characteristics from developed countries that need to be investigated as moderating factors instead of only as confounders, as socioeconomic condition was an indicator of health inequalities [20] and of PA engagement [21] and time spent in SB [22] in adolescents. The stratification of socioeconomic status and parental sex provides an in-depth analysis of the occurrence of different associations of parent-child PA and SB, leading to specific decision making in public policies and interventions for family health promotion. In this sense, the present study aimed to investigate the association of parent-child PA and SB according to parental socioeconomic strata and sex for both adolescents and their parents.

## Methods

### Sample

The sample was composed of 1231 adolescents aged 12–17 years (515 boys and 716 girls) from the six largest schools in the central region of the city of Londrina (~ 500,000 inhabitants), located in the southern region of Brazil. To calculate the sample size, a relationship between sedentary behaviour of parents and children of r = 0.12 [22], a power of 80%, and an alpha error of 5% were considered. Predicting possible sample losses, 30% were added to the sample calculation, resulting in a minimum number of 706 adolescents.

Parents or guardians received a questionnaire about their lifestyle habits (PA and SBs) and education level. In total, 1202 mothers and 871 fathers answered the questions. Parents or guardians of the adolescents signed a consent form authorizing their participation in the study.

### Ethical approval

All methods were carried out in accordance with relevant guidelines and regulations, along with an ethical approval statement and informed consent to participate. The ethics committee of the State University of Londrina approved this study (n°203/10), and all study procedures were performed following the Declaration of Helsinki. Adolescents could quit the study at any time without negative consequences. There were no incentives for participation.

### Physical activity and anthropometric measures

The PA questionnaire used in this study was the instrument by Baeke et al. [23]. This questionnaire is validated for adolescents [24] and Brazilian adults [25] and consists of different domains; sports and/or physical exercise (1 question stratified into 3 questions considering the intensity, weekly time [in hours], and previous time of practice [in months]), and leisure and commuting PA, considering active commuting as walking or cycling during occupational activities such as going to school, work, or shopping. Therefore, the leisure time PA and commuting PA of adolescents and their parents were used in this study.

Height was measured with a portable stadiometer (accuracy of 0.1 cm and maximum length of 200 cm). Weight was measured with an electronic scale (accuracy of 0.1 kg and maximum capacity of 150 kg) of Plena® (Acqua model, São Paulo, SP, Brazil).

### Sedentary behaviour

The domains of SB were assessed by the number of hours per week of television (TV) and computer use by the parents of adolescents. These types of SB domains have been used in studies that evaluated the SB of adolescents [26] and adults [27].

### Economic classification

The economic classification was evaluated using an instrument from the “Associação Brasileira de Empresas e Pesquisa”, [28] which assesses the economic classification in Brazil according to education and consumer goods. The sum of points between the questions generates a socioeconomic classification, and the higher the score, the better the classification. Economic classes A1 and A2 were considered as high, B1 and B2 as medium, and ≤ C1 as low economic class.

### Statistical analysis

The Kolmogorov-Smirnov test was performed to verify the normality of the data. Sample characterization variables are described as mean and standard deviation, compared by economic classification with Analysis of Variance (ANOVA) using the Bonferroni post-hoc test, with the effect size expressed as eta-squared (ES-r), as follows: ES-r < 0.06 (small effect-size), ES-r ≥ 0.06 and < 0.140 (moderate effect-size), and ES-r ≥ 0.140 (high effect-size). Pearson correlation coefficients were adopted to analyze the strength of the bivariate relationships as small (< 0.3), medium (≥ 0.3 and < 0.5), and large (≥ 0.5) [29]. The association between different domains of PA (leisure-time PA and commuting PA) and SB (TV and computer) of adolescents with the different domains of PA and SB of their parents was analyzed by linear regression adjusted by adolescents’ age, and parents’ age and education level, with the effect size expressed as r-squared (R²). The confidence interval adopted was 95% and a statistical significance of 5%. The statistical package used was SPSS.

## Results

The sample of the present study was composed of 1231 adolescents, 1202 mothers and 871 fathers. Table 1 shows the sample characterization variables considering the socioeconomic classification. High Economic Class adolescents were heavier (Post hoc P = 0.015), and practiced more PA in leisure (Post hoc P = 0.011) than Low Economic Class; Medium Economic Class adolescents were taller (Post hoc P = 0.002), practiced more PA in leisure (Post hoc P = 0.032), and spent more hours each week using the computer (Post hoc P < 0.001) than Low Economic Class.

**Table 1:** Sample characterization (n = 1231 adolescents; 871 fathers and 1202 mothers).

	High economic class (n = 128)^a^	Medium economic class (n = 352)^b^	Low economic class (n = 751)^c^	ANOVA	Post hoc p-value			ES
	Mean (SD)	Mean (SD)	Mean (SD)	p-value	a x b	a x c	b x c	
**Adolescents**	(n = 128)	(n = 352)	(n = 751)					
Age (years)	15.48 (1.06)	15.55 (1.05)	15.57 (1.08)	0.652	0.999	0.999	0.999	0.001
Weight (kg)	62.81 (15.81)	60.88 (12.86)	59.30 (12.57)	**0.008**	0.453	0.015	0.185	0.006
Height (cm)	167.99 (9.33)	167.63 (9.20)	165.94 (9.04)	**0.001**	0.999	0.158	**0.002**	0.011
Leisure time PA (B. score)	4.22 (2.25)	3.95 (2.43)	3.56 (2.30)	**0.002**	0.817	0.011	**0.032**	0.010
Commuting PA (B. score)	2.36 (0.64)	2.30 (0.67)	2.30 (0.69)	0.576	0.999	0.884	0.999	0.001
TV (hours/week)	15.04 (9.09)	15.09 (9.73)	16.90 (9.92)	0.053	0.999	0.136	0.231	0.005
Computer (hours/week)	19.96 (10.49)	21.34 (10.83)	18.06 (11.13)	**< 0.001**	0.680	0.217	**< 0.001**	0.017
**Fathers**	(n = 101)	(n = 262)	(n = 508)					
Age (years)	47.25 (7.30)	45.92 (7.06)	45.38 (7.65)	0.066	0.387	0.065	0.999	0.006
Weight (kg)	82.74 (12.81)	83.39 (15.54)	81.13 (14.20)	**0.036**	0.999	0.927	**0.033**	0.008
Height (cm)	173.99 (6.80)	173.68 (7.08)	172.69 (6.97)	0.083	0.999	0.272	0.207	0.006
Leisure time PA (B. score)	3.71 (2.42)	3.03 (2.12)	2.56 (1.78)	**< 0.001**	**0.010**	**< 0.001**	**0.006**	0.035
Commuting PA (B. score)	2.20 (0.61)	2.16 (0.74)	2.22 (0.74)	0.680	0.999	0.999	0.999	0.001
TV (hours/week)	24.43 (10.96)	24.22 (11.30)	21.82 (11.10)	**0.006**	0.999	0.097	**0.015**	0.012
Computer (hours/week)	29.87 (15.61)	29.29 (15.93)	26.21 (15.43)	**0.010**	0.999	0.096	**0.029**	0.010
**Mothers**	(n = 125)	(n = 342)	(n = 735)					
Age (years)	44.50 (6.59)	43.22 (6.92)	43.14 (7.39)	0.142	0.259	0.152	0.999	0.003
Weight (kg)	68.71 (12.91)	67.65 (12.63)	68.06 (12.94)	0.722	0.999	0.999	0.999	0.001
Height (cm)	163.28 (6.00)	161.73 (6.10)	160.86 (6.64)	**< 0.001**	0.064	**< 0.001**	0.117	0.014
Leisure time PA (B. score)	2.50 (1.64)	2.31 (1.50)	2.12 (1.32)	**0.007**	0.603	**0.016**	0.116	0.007
Commuting PA (B. score)	2.10 (0.65)	2.09 (0.62)	2.21 (0.67)	**0.023**	0.603	**0.016**	0.116	0.006
TV (hours/week)	24.11 (10.22)	22.36 (10.58)	12.89 (11.20)	0.108	0.382	0.108	0.999	0.004
Computer (hours/week)	28.96 (14.41)	26.90 (14.82)	26.27 (15.53)	0.185	0.585	0.206	0.999	0.010

Notes: Numerical variables are reported as the mean ± standard deviation. Abbreviations: ES: Effect size; B. score = Baecke score. Bold values denote statistical significance. Post hoc p-value/superscript letters denote a higher significant difference between groups: a (high economic class), b (medium economic class), c (low economic class) with P < 0.050.

Fathers from the High Economic Class practiced more PA in leisure than Medium Economic Class (Post hoc P = 0.010) and Low Economic Class (Post hoc P < 0.001); fathers from the Medium Economic Class were taller (Post hoc P = 0.033), practiced more PA in leisure (Post hoc P = 0.006), watched more TV (Post hoc P = 0.015), and spent more hours each week using the computer (Post hoc P = 0.029) than Low Economic Class.

Mothers from High Economic Class were taller (Post hoc P < 0.001), practiced more leisure PA (Post hoc P = 0.016), and had a higher score for active commuting (Post hoc P = 0.016) than Low Economic Class; mothers from the Medium Economic Class had a higher score for active commuting (Post hoc P = 0.016) than Low Economic Class.

Correlations analyses of the different domains of PA and SB between boys and girls and their parents are presented in Tables 2 and 3, respectively. In boys, leisure (P < 0.001 and P = 0.028) and commuting PA (P = 0.004 and P = 0.001) were positively related with their fathers’ respective domains only in the low socioeconomic class, while considering mothers, commuting PA of boys was related to their mothers’ commuting PA in High (P = 0.034) and Medium (P = 0.002) socioeconomic classes, and TV time was positively related with their fathers’ respective domains only in the low socioeconomic class (P < 0.001). In girls, leisure and commuting PA were positively related with fathers’ respective PA activity (P = 0.001) and TV time (P < 0.001) in Low Economic Class. Commuting PA was positively related with their mothers’ respective domains in the High and Medium Economic Classes (P < 0.001 for both), in Low Economic Class, leisure time PA, TV time, and time on Computer were positively related with their mothers’ respective domains (P < 0.001 for all) and Commuting PA (P = 0.001).

**Table 2:** Correlation between different domains of physical activity and sedentary behaviour in boys and their parents (n = 1231 adolescents; 871 fathers and 1202 mothers)

Boys	Fathers			Mothers		
	r (95% CI)	P	Correlation strength	r (95% CI)	P	Correlation strength
**High economic class**						
Leisure time PA	0.26 (0.19; 0.32)	0.058	-	0.15 (0.09; 0.20)	0.228	-
Commuting PA	0.11 (0.04; 0.17)	0.434	-	**0.27 (0.27; 0.32)**	**0.034**	Small
TV	0.02 (-0.04; 0.08)	0.871	-	0.12 (0.06; 0.17)	0.338	-
Computer	0.06 (-0.01; 0.12)	0.665	-	0.15 (0.09; 0.20)	0.242	-
**Medium economic class**						
Leisure time PA	0.11 (0.04; 0.17)	0.216	-	0.08 (0.02; 0.13)	0.260	-
Commuting PA	0.16 (0.09; 0.22)	0.073	-	**0.25 (0.19; 0.30)**	**0.002**	Small
TV	0.02 (-0.04; 0.08)	0.786	-	0.18 (0.12; 0.23)	0.210	-
Computer	0.04 (-0.02; 0.10)	0.638	-	0.09 (0.03; 0.14)	0.245	-
**Low economic class**						
Leisure time PA	**0.27 (0.20; 0.33)**	**< 0.001**	Small	**0.14 (0.08; 0.19)**	**0.028**	Small
Commuting PA	**0.21 (0.14; 0.27)**	**0.004**	Small	**0.23 (0.17; 0.28)**	**0.001**	Small
TV	**0.38 (0.32; 0.43)**	**< 0.001**	Medium	0.09 (0.03; 0.14)	0.101	-
Computer	0.12 (0.05; 0.18)	0.110	-	0.05 (-0.01; 0.10)	0.451	-

Notes: The data are presented as correlation (r), Bold values denote statistical relation/significance with P < 0.050.

**Table 3:** Correlation between different domains of physical activity and sedentary behaviour in girls and their parents (n = 1231 adolescents; 871 fathers and 1202 mothers)

Girls	Fathers				Mothers		
	r (95% CI)	P	Correlation strength	r (95% CI)		P	Correlation strength
**High economic class**							
Leisure time PA	0.21 (0.14; 0.27)	0.145	-	0.18 (0.12; 0.23)		0.143	-
Commuting PA	**0.33 (0.26; 0.38)**	**0.020**	Medium	**0.51 (0.46; 0.55)**		**< 0.001**	Large
TV	0.18 (0.11; 0.24)	0.202	-	0.05 (-0.07; 0.10)		0.684	-
Computer	0.16 (0.09; 0.22)	0.255	-	0.12 (0.06; 0.17)		0.342	-
**Medium economic class**							
Leisure time PA	**0.28 (0.21; 0.34)**	**< 0.001**	Small	0.12 (0.06; 0.17)		0.099	-
Commuting PA	**0.30 (0.23; 0.35)**	**< 0.001**	Medium	**0.32 (0.26; 0.37)**		**< 0.001**	Medium
TV	0.13 (0.06; 0.19)	0.114	-	0.14 (0.08; 0.19)		0.068	-
Computer	0.06 (-0.06; 0.12)	0.447	-	0.07 (0.01; 0.12)		0.339	-
**Low economic class**							
Leisure time PA	0.11 (0.04; 0.17)	0.061	-	**0.30 (0.24; 0.35)**		**< 0.001**	Medium
Commuting PA	**0.28 (0.21; 0.34)**	**0.001**	Small	**0.28 (0.22; 0.33)**		**0.001**	Small
TV	**0.30 (0.23; 0.35)**	**< 0.001**	Medium	**0.31 (0.25; 0.36)**		**< 0.001**	Medium
Computer	0.10 (0.03; 0.16)	0.285	-	**0.27 (0.27; 0.32)**		**< 0.001**	Small

Notes: The data are presented as correlation (r), Bold values denote statistical relation/significance with P < 0.050.

Table 4 presents the associations between the domains of PA and SB of boys and their parents by sex and socioeconomic status, adjusted by confounders. In boys, leisure time PA was associated with their fathers’ leisure time PA in both High (β = 0.23 [0.06; 0.45]) and Low Economic Classes (β = 0.31 [0.14; 0.48]) while in commuting PA, there was an association with their fathers’ commuting PA only in Low Economic Class (β = 0.21 [0.06; 0.35]). On the other hand, considering mothers, boys’ commuting PA was associated with mother’s commuting PA in all economic classes (β = 0.15 to 0.29). Lastly, with respect to sedentary behaviour domains, TV time in boys was associated with TV time of their fathers in the Low Economic Class (β = 0.13 [0.02; 0.25]) and with TV time of their mother in the Medium Economic Class (β = 0.13 [0.00; 0.27]).

**Table 4:** Association between different domains of physical activity and sedentary behaviour in adolescents and their parents, according to sex and socioeconomic status (adolescents n = 1231; mothers n = 1202; fathers n = 871)

	PA domains						Sedentary behaviour domains					
	Leisure time PA			Commuting PA			TV			Computer		
	β (95% CI)	p-value	R^2^	β (95% CI)	p-value	R^2^	β (95% CI)	p-value	R^2^	β (95% CI)	p-value	R^2^
**Boys**												
**High** **economic class**												
Father	**0.23 (0.06; 0.45)**	**0.044**	0.09	0.12 (-0.22; 0.46)	0.472	0.02	0.10 (-0.11; 0.32)	0.353	0.10	-0.45 (-1.95; 1.05)	0.551	0.04
Mother	0.22 (-0.10; 0.55)	0.178	0.04	**0.28 (0.03; 0.53)**	**0.027**	0.09	0.09 (-0.14; 0.32)	0.444	0.02	1.10 (-0.27; 2.49)	0.114	0.17
**Medium** **economic class**												
Father	0.13 (-0.05; 0.32)	0.167	0.03	0.14 (-0.03; 0.31)	0.110	0.04	0.07 (-0.07; 0.23)	0.313	0.04	-0.14 (-1.02; 0.72)	0.735	0.04
Mother	0.13 (-0.10; 0.37)	0.259	0.01	**0.29 (0.11; 0.47)**	**< 0.001**	0.07	**0.13 (0.00; 0.27)**	**0.046**	0.05	0.48 (-0.28; 1.24)	0.218	0.03
**Low** **economic class**												
Father	**0.31 (0.14; 0.48)**	**< 0.001**	0.10	**0.21 (0.06; 0.35)**	**0.005**	0.05	**0.13 (0.02; 0.25)**	**0.022**	0.08	0.64 (-0.02; 0.13)	0.059	0.03
Mother	0.19 (-0.02; 0.41)	0.083	0.04	**0.15 (0.02; 0.28)**	**0.020**	0.03	0.01 (-0.08; 0.11)	0.789	0.08	0.27 (-0.33; 0.87)	0.382	0.01
**Girls**												
**High** **economic class**												
Father	0.14 (-0.11; 0.40)	0.269	0.16	0.28 (-0.00; 0.56)	0.051	0.12	0.18 (-0.13; 0.48)	0.256	0.10	**1.72 (0.05; 3.39)**	**0.043**	0.16
Mother	0.25 (-0.07; 0.58)	0.123	0.14	**0.45 (0.21; 0.69)**	**< 0.001**	0.29	-0.04 (-0.28; 0.20)	0.748	0.07	0.81 (-0.51; 2.14)	0.223	0.08
**Medium** **economic class**												
Father	**0.27 (0.11; 0.42)**	**0.001**	0.10	**0.21 (0.09; 0.32)**	**0.001**	0.11	0.04 (-0.11; 0.19)	0.607	0.01	0.35 (-0.48; 1.19)	0.404	0.03
Mother	0.18 (-0.02; 0.39)	0.085	0.04	0.27 (0.15; 0.39)	0.085	0.11	**0.15 (0.01; 0.29)**	**0.029**	0.04	0.50 (-0.28; 1.28)	0.210	0.04
**Low** **economic class**												
Father	0.11 (-0.01; 0.22)	0.061	0.02	**0.16 (0.06; 0.24)**	**0.001**	0.05	**0.28 (0.18; 0.39)**	**< 0.001**	0.12	0.47 (-0.11; 1.04)	0.118	0.02
Mother	**0.38 (0.25; 0.50)**	**< 0.001**	0.08	**0.23 (0.14; 0.32)**	**< 0.001**	0.07	**0.25 (0.17; 0.32)**	**< 0.001**	0.09	**0.57 (0.12; 1.03)**	**0.014**	0.02

Adjusted by adolescent’s age, parent’s age, and parent’s education

*Note: Each domain association analysis analyzed separately; ex: father’s leisure time PA and their sons’ and daughters’ leisure time PA; Mother’s TV use and their sons’ and daughters’ TV use, , use, Bold values denote statistical association/significance with P < 0.050.

In girls, leisure time was associated with their fathers’ leisure time PA only in Medium Economic Class (β = 0.27 [0.11; 0.42]), while in commuting PA there was an association with their fathers’ commuting PA in Medium (β = 0.21 [0.09; 0.32]) and Low Economic Class (β = 0.16 [0.06; 0.24]). In associations with mothers, girl’s leisure time PA was associated with mother’s leisure time PA in Low Economic Class (β = 0.38 [0.25; 0.50]) while commuting PA was associated with mother’s commuting PA in High (β = 0.45 [0.21; 0.69]) and Low Economic Class (β = 0.23 [0.14; 0.32]). Considering sedentary behaviour domains, TV time in girls was associated with TV time of their fathers only in Low Economic Class (β = 0.28 [0.18; 0.39]), while computer use was associated with their fathers’ computer use only in High Economic Class (β = 1.72 [0.05; 3.39]). On the other hand, girls’ TV time was associated with mother’s TV time in Medium (β = 0.15 [0.01; 0.29]) and Low Economic Classes (β = 0.25 [0.17; 0.32]), and with use of computer only in Low Economic Class (β = 0.57 [0.12; 1.03]).

## Discussion

This cross-sectional study aimed to analyze the associations between PA and SB of adolescents and their parents according to sex and socioeconomic status. The main results showed that socioeconomic status and sex of the parents are important determinants that can affect the association between PA (leisure and commuting) and SB (TV and computer use) of adolescents and their parents.

The association between PA and SB of children/adolescents and their parents has been extensively analyzed in the literature. A study carried out in Australia for example, observed a positive relationship between the practice of physical activity between parents and children [30]. In a European population, a cross-sectional study involving almost 900 children and adolescents showed that PA of both parents (mothers and fathers) is influential for children’s sport participation [31]. Lastly, similar results were observed in Brazil in a recent study, in which sedentary behaviour and physical activity of parents were associated with their children’s sedentary behaviour and physical activity [22]. These studies are consistent with our findings, corroborating the hypothesis that children tend to follow their parents’ behaviour as they feel encouraged and positive cognitions about the practice of physical activity are created, [31–35], besides highlighting the determinant effect of parent and child sex in the association.

Our study advances the knowledge, demonstrating that economic status can affect this association. Leisure PA, for example, seems to be more affected by economic class than commuting physical activity in which there was an association between mothers and children (boys and girls) for all economic classes (except medium class in girls). Sex-based parenting roles still mostly attribute looking after the children to mothers [18], which could explain the higher maternal influence on children’s PA, mainly on weekdays, [18] when most commuting activities occur (e.g., to work/school) and extracurricular activities. Thus, our findings, in addition to being in accordance with the literature, demonstrate that this influence is independent of the mother’s economic status and, consequently, of possible inequalities.

Interestingly, the present study observed significant associations between parent-child commuting PA for both parents only in adolescents of low economic status. It is known that the influence of economic status on PA can be explained by different aspects, such as social, biological, and behavioural factors, [14, 36] that include differences in access to sports facilities, free time, safety for PA practice, coach support, and obesity [14, 36, 37]. Thus, it is possible that parents with a lower income do not commonly own motor vehicles for commuting, which would imply greater active commuting (walking or cycling) for them and their children. However, as significant associations of commuting PA were observed even in high economic class, further investigations are important to clarify whether active commuting is due to a free choice for an active lifestyle or to economic limitations. In addition, leisure time among low-income parents and children is usually linked to the use of public facilities, which indirectly increases PA in this group and can support, in part, the stronger association observed in the present study for the medium-to-low economic classes.

Regarding SB, the parent’s influence on their children seems to occur mostly in the lower and medium economic classes, especially TV viewing. Previous research reported that the amount of TV watched per week is higher in older children with a low economic status [36], with family economic status being negatively related with SB [38], agreeing with our findings. Parents with a lower economic condition, usually present less education and, consequently, may have less access to information about the negative risks of SB [39]. In addition, public facilities for leisure PA (e.g., parks and recreation centres) are often observed in neighbourhoods of people with higher economic conditions [40, 41]. These inequalities can lead parents from low and medium economic classes to opt for family activities at home as a leisure option, such as watching TV, which influences their children to adopt the same habit. The parent-child computer use association occurred only among girls, with associations with fathers from high economic class and mothers from low economic class. Although a positive association of computer use with employment status and educational level has been previously reported, [42] the dynamic advance of technology since then allows a wider range of functions by computers. In this sense, the findings of the present study require further investigation about the social contextual factors of computer use at home and outside home, as this behaviour may be related to occupational activities (i.e., study or working tasks) or entertainment purposes (i.e., social media, electronic games, watching movies and series) of parents and can be differently associated with the purpose and amount of computer use by their children.

Despite the innovation and good sample size, the study has limitations that need to be recognized. Firstly, the cross-sectional study design precludes a causal interpretation of the results. Second, the use of questionnaires to measure PA and SB may present recall bias, being considering as an indirect method (self-report). Furthermore, the study did not consider other forms of SB, such as mobile phone use and reading a book. Lastly, due to the smaller number of fathers, the paternal association modelling was tested in only 70.7% of the sample.

## Conclusion

In summary, in addition to sex, the results from this study suggest that economic status is an important determinant of the association between time spent on PA and/or SB of Brazilian children and their parents. The mother’s influence seems to be more determinant, while low-income parents tend to have a greater influence on the PA and SB of their children. These findings reinforce the importance of awareness and application of public policies aimed at reducing the social inequalities between the economic classes, which affect lifestyle habits in both parents and children.

## Data Availability

The data collected at the State University of Londrina and analyzed during this study are stored by the authors upon authorization by the leader of the Laboratory of InVestigation in Exercise (LIVE). Additionally, data from this study, can be requested from the corresponding author.

## List of abbreviations

PA: Physical activity
TV: Television
ANOVA: Analysis of variance
SB: Sedentary behaviour
LIVE: Laboratory of InVestigation in Exercise
